# Editorial: Novel molecular targets in cancer therapy

**DOI:** 10.3389/fonc.2025.1766079

**Published:** 2026-01-14

**Authors:** Sandip Patil, Rehan Khan, Nemat Ali

**Affiliations:** 1 Department of Haematology and Oncology, Shenzhen Children’s Hospital, Shenzhen, China; 2 Paediatric Research Institute, Shenzhen Children’s Hospital, Shenzhen, China; 3 Public Health Research Institute, Rutgers New Jersey Medical School, Rutgers Biomedical and Health Sciences, Newark, NJ, United States; 4 Department of Pharmacology and Toxicology, College of Pharmacy, King Saud University, Riyadh, Saudi Arabia

**Keywords:** biomarker discovery, molecular oncology, multi-omics profiling, therapeutic targets, tumour microenvironment

Rapid advances in molecular oncology, multi-omics profiling, and high-resolution tumour mapping continue to reshape our understanding of cancer initiation, progression, and therapeutic resistance. As cancer remains a major global health burden, the identification of novel molecular targets has become central to the development of precise, durable, and context-specific interventions. The Research Topic Novel Molecular Targets in Cancer Therapy provides a dedicated platform for showcasing emerging biomarkers, mechanistic discoveries, translational targets, and early-stage therapeutic strategies across diverse malignancies. The articles collected here collectively illustrate how integrative molecular approaches can uncover actionable vulnerabilities, thereby refining prognostic and therapeutic frameworks. A major focus of this Research Topic is the use of genomic and transcriptomic tools to identify biomarkers that not only predict clinical outcomes but may also serve as direct therapeutic targets. In colorectal cancer liver metastasis, a comprehensive single-cell transcriptomic study constructed a prognostic model and dissected macrophage migration inhibitory factor (MIF)–related signalling, revealing cellular heterogeneity and regulatory dependencies that may guide personalized therapy (Cao et al.). Downregulation of SALL1 in clear cell renal cell carcinoma was found to correlate with poor prognosis and alterations in the immune cell landscape, suggesting that SALL1 may have dual relevance as both a prognostic biomarker and a modulator of tumour–immune interactions (Qu et al.). Expanding beyond tumour-specific analysis, a pan-cancer study of SLC7A11 highlighted its prognostic significance in hepatocellular carcinoma and reinforced the therapeutic importance of redox regulation, as elevated SLC7A11 expression was linked to adverse clinical outcomes (Zhang et al.). In glioblastoma, MEX3A was identified as a diagnostic and independent prognostic biomarker, offering a promising foundation for therapeutic targeting in one of the most treatment-resistant cancers (Bufalieri et al.). Together, these contributions emphasize the value of molecular stratification in exposing targetable liabilities and improving outcome prediction. Beyond identifying individual prognostic biomarkers, this Research Topic highlights mechanistic and systems-level approaches for molecular subtyping and immune-related therapeutic discovery. For instance, in Wilms tumour, a study integrating immune gene signatures delineated NK cell–associated molecular subtypes. This approach not only revealed prognostic differences but also nominatedHS2ST1 as a potential biomarker. Significantly, the work identified TGX-221 as a potent candidate therapeutic agent for patients with poor survival signatures, underscoring the direct translational impact of this immune-driven molecular classification (Hong et al.). These findings align with the growing recognition that the tumour immune microenvironment is integral not only to cancer progression but also to therapy responsiveness. By leveraging immune-related transcriptional markers, the study provides a framework for refining immunotherapeutic strategies in paediatric renal cancers. Therapeutic innovation is also represented through early-phase clinical evaluation and detailed assessment of emerging anti-cancer modalities. A phase I/II clinical investigation of L-DOS47, an antibody–enzyme conjugate designed to target the tumour microenvironment in advanced non–small cell lung cancer (NSCLC), demonstrated a favourable safety profile and clinical feasibility. This work highlights the ongoing evolution of antibody-based therapeutics and validates the translational potential of pharmacologic strategies aimed at modulating tumour pH and metabolic milieu (Ramlau et al.). Complementing this, an authoritative review on TROP2 consolidated current knowledge on its role as an oncogenic driver in lung cancer, summarizing therapeutic approaches including antibody–drug conjugates and monoclonal antibodies targeting this clinically relevant cell-surface molecule. (TROP2: A promising target for lung cancer therapy (). Together, these contributions reflect the therapeutic progression from target identification to early-stage clinical application.

Expanding beyond protein-coding genes, recent research highlights the importance of post-transcriptional and post-translational regulators in shaping tumour behaviour. While non-coding RNAs and other regulatory molecules are increasingly recognized as critical contributors to oncogenesis, an illustrative contribution within this Research Topic specifically focuses on glycosylation-related mechanisms. The study systematically profiled glycosyltransferase dysregulation in NSCLC and identified several enzymes implicated in tumour progression and immune modulation (Liu et al.). These findings demonstrate how aberrant glycosylation influences cell–surface interactions, receptor activation, and microenvironmental signalling, thereby expanding the spectrum of actionable molecular targets beyond classical oncogenes (Alaseem et al.). The studies included in this Research Topic underscore a central theme: the future of cancer therapy will rely on three pillars: molecular precision, therapeutic diversification, and integration of multi-omics approaches. Whether through identifying prognostic biomarkers, defining immune-related subtypes, or uncovering novel regulatory pathways such as glycosylation networks progress in oncology increasingly depends on detailed mechanistic insight coupled with translational rigor. These contributions highlight the expanding repertoire of potential therapeutic targets and reinforce the importance of systems-level investigation in refining precision oncology.

We extend our sincere appreciation to all authors, reviewers, and contributors for their engagement and scientific commitment. The discoveries highlighted in this Research Topic broaden the current understanding of molecular targets in cancer and support ongoing efforts to develop the next generation of individualized, effective cancer therapies.

## Conclusion

The Novel Molecular Targets in Cancer Therapy Research Topic offers a robust snapshot of contemporary efforts to refine and expand molecularly guided cancer treatment. The breadth of contributions from biomarker identification and prognostic modelling to clinical translation and therapeutic review demonstrates both the progress made and the challenges ahead in targeting cancer at the molecular level. We thank all authors and reviewers for their valuable contributions, which collectively advance the field toward more precise, effective, and personalized cancer therapies.

